# Effects of Teriparatide on Bone Mineral Density and Prevention of Fragility Fractures in Saudi Arabian Subjects with Osteoporosis: A Two-Year Clinical Study from a Single Center

**DOI:** 10.1155/2022/3779745

**Published:** 2022-10-25

**Authors:** Mir Sadat-Ali, Abdallah Al-Omran, Khalid AlTabash, Dakheel A. AlDakheel, Salma Elansassy, Tarek Hegazi, Mona Al Muhaish

**Affiliations:** ^1^Department of Orthopaedic Surgery, College of Medicine, Imam AbdulRahman Bin Faisal University Dammam and King Fahd Hospital of the University, AlKhobar, Saudi Arabia; ^2^Department of Pharmacy, College of Medicine, Imam AbdulRahman Bin Faisal University Dammam and King Fahd Hospital of the University, AlKhobar, Saudi Arabia; ^3^Department of Radiology, College of Medicine, Imam AbdulRahman Bin Faisal University Dammam and King Fahd Hospital of the University, AlKhobar, Saudi Arabia

## Abstract

**Design:**

A prospective study was conducted. *Setting*. This study took place at King Fahd Hospital of the University, Imam AbdulRahman Bin Faisal University, Dammam, Saudi Arabia. *Primary and Secondary Outcomes*. The study aimed to evaluate changes in BMD and prevention of fragility fractures.

**Materials and Methods:**

We followed up 439 patients who were prescribed teriparatide at the King Fahd Hospital of the University, AlKhobar, and 415 (94.5%) patients completed a 24-month teriparatide course. The data gathered before starting medication were age, sex, previous therapy, history of fractures, and other diseases like diabetes mellitus, hypertension, and cardiac disease. At the time of the final assessment after 24 months, a history of fractures if any during the treatment was collected and a DXA scan was done.

**Results:**

A total of 415 patients were followed up for 2 years. Three hundred and sixty-five patients (87.9%) were females, and the rest were males. The average age was 68.21 ± 17.6 years. Two hundred and forty-eight patients (59.8%) were treatment naïve, and 167 (40.2%) were on treatment for osteoporosis. Twenty patients (4.8%) sustained fracture on treatment. The pretreatment DXA showed that the mean hip T-score was −3.1 ± 0.79, and after completion of the treatment, it was −1.5 ± 0.62 (*P* < 0.001), while the T-score of the lumbar spine was 4.4 ± 0.86 versus −3.2 ± 0.87 (*P* < 0.001). Seventeen (4.09%) had fractures while on teriparatide treatment. The mean significant gain (MSG) for BMD for the hip was 0.095 g/cm^2^, and for the lumbar spine, it was was 0.109 g/cm^2^ with *P* < 0.001 at 95% CI.

**Conclusions:**

Our study shows that 94.5% completed the treatment duration, and there was an increase in the BMD. The decrease in T-scores of the lumbar spine and hip was significant so was the reduction in the number of fractures.

## 1. Introduction

Osteoporosis is an ageing disease which is due to the imbalance in bone formation and bone resorption. This is most commonly seen in women in early menopause due to lack of estrogen. The cost of osteoporosis management and its complications like fractures is tremendous. It was estimated that in the United States of America, osteoporosis affected 10 million adults, with a cost of $22 billion in 2002. [1] In Saudi Arabia, it was assessed that the cost of osteoporosis-related fractures could reach Saudi Riyals 35 billion by 2050. [2] Osteoporosis still remains a silent disease, and in the majority of men and women, a fracture indicates the presence of osteoporosis. In a recent study, only 33% of patients with osteoporosis have received bisphosphonates and others have been untreated or treated with calcium and vitamin D [3]. The under diagnosis and under treatment even after a fragility fracture are universal. In the US only 15% of patients with a fragility fracture were diagnosed with osteoporosis, while in Europe, the story is not different, where 72% of women with a fracture had no diagnosis of osteoporosis. [4, 5] Unfortunately, patients after the first fragility fracture are not treated appropriately, resulting in a second fracture, which is more devastating than the first one. Hence, it is imperative to treat fragility fractures to recognize and prevent a second fracture. [6, 7] To prevent the first or second fracture, high-risk patients need to be on appropriate therapy for osteoporosis; and one such treatment is an anabolic agent.

Recent crucial data in randomized trials have shown that anabolic agents reduce both vertebral and nonfractures quicker than other antiresorptive therapies. [8] Since all Saudi Arabian patients receive free treatment and medications, this study was undertaken to assess the effects of teriparatide on bone mineral density and prevention of fragility fractures in Saudi Arabian subjects with osteoporosis.

## 2. Material and Methods

The study was approved by the Institutional Review Board of Imam AbdulRahman Bin Faisal University Dammam, vide number 2019-01-105. Informed signed consent was obtained from all patients receiving treatment who were part of the study. All 439 patients were followed up who were prescribed teriparatide at the King Fahd Hospital of the University, AlKhobar, and 415 (94.5%) patients completed a 24-month teriparatide course. The data gathered before starting the medication were age, sex, previous therapy, history of fractures, and other diseases like diabetes mellitus, hypertension, and cardiac disease. The indication of anabolic therapy was patients with fragility fractures, with a T-score of <−3.5 and those with a family history of fragility fractures. All patients had a DXA scan performed before the treatment and 24 months later. Hologic Discovery Model A (S/N87624), software version 13.4.2, Marlborough, MA 01752, USA, was used for pretreatment and post-treatment DXA scans. All patients had a repeat DXA scan after 24 months of the treatment. Vitamin D and calcium were given to all patients so that 25OHD remained ≥30 ng/mL. At the time of final assessment after 24 months, any fractures during this treatment period was entered in the data base. Patient compliance was regularly monitored by the hospital nurse and the Eli Lilly support staff. The data were entered in the database and analyzed using SPSS Ver 24. The data were expressed as a mean ± SD. Statistically significant differences between the different groups were determined using Student's *t*-test, and *P* < 0.05 was considered to be significant and was ascertained at a CI of 95%.

## 3. Results

**Figure 1:**
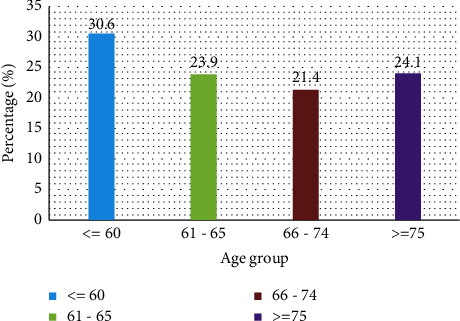
Percentage for the age group (*n* = 415).

**Figure 2:**
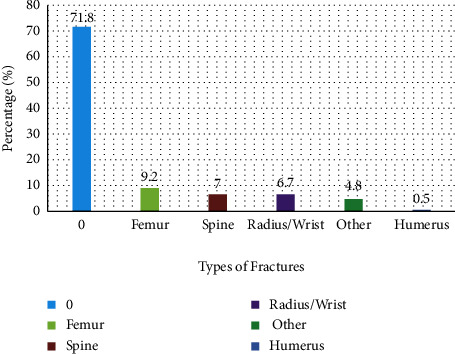
Percentage for types of fractures (*n* = 415).

Four hundred and thirty-nine patients were prescribed teriparatide at the King Fahd Hospital of the University, AlKhobar, and 415 (94.5%) patients completed a 24-month teriparatide course. A total of 415 patients were followed up for 2 years. Three hundred and sixty-five patients (87.9%) were females, and the rest were male patients. The average age was 68.21 ± 17.6.  Figure 1 shows the age range of all patients. Two hundred and forty-eight patients (59.8%) were treatment naïve, and 167 patients (40.2%) were on treatment for osteoporosis (Table 1). One hundred and seventeen (28.2%) presented with fractures, and 298 (71.8%) had no fractures (Figure 2). Twenty (4.8%) patients sustained fracture on treatment, and 97 (23.4%) had fractures while not on treatment (*P* < 0.001). The pretreatment DXA showed that the mean hip T-score was −2.0 ± 0.79, and after completion of the treatment, it was −1.5 ± 0.62 (*P* < 0.001), while the T-score of the lumbar spine was −4.4 ± 0.86 versus −3.2 ± 0.87 (*P* < 0.001 (Table 2). The mean significant gain (MSG) for BMD for the hip was 0.095 g/cm^2^, and for the lumbar spine, it was 0.109 g/cm^2^ with *p* < 0.001 at 95% CI.

**Figure 3:**
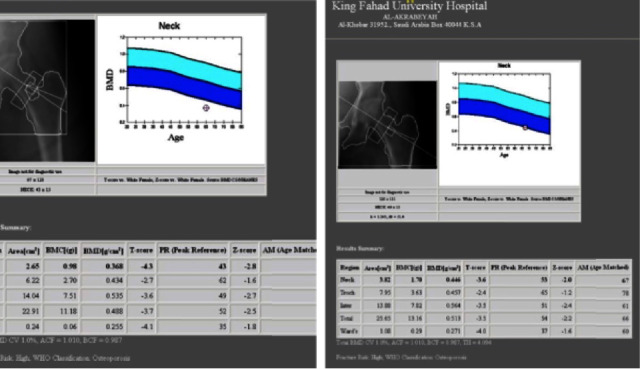
Pretreatment and post-treatment DXA scans of the hip.

**Figure 4:**
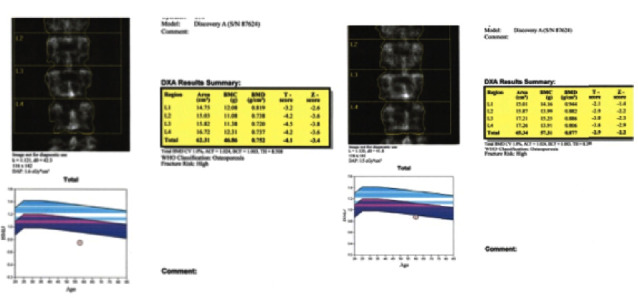
Pretreatment and post-treatment DXA scans of the lumbar spine.

Figures 3 and 4 show the BMD gain pretretment and post-treatment of the hips and lumbar spine. Seventeen (4.09%) had fractures while on teriparatide treatment. There were no adverse reactions reported which made to stop the treatment.

## 4. Discussion

Our study has shown that the overall change in BMD was 20% in both the hip and lumbar spine, which was in contrast to the earliest report of 6 and 13 percentage points. [9] Miyauchi et al. [10] reported that after 24 months of teriparatide use in their patients, the increase in BMD was observed to be 13.42%. Anagnostis et al. [11] reported that in patients with lactation-related severe osteoporosis treated with teraperatide showed BMD an increase of 13.1% at the lumbar spine at 12 months and 19.1% after 24 months, respectively.In our patients as reported, the overall increase in BMD was 20%. The reasons for this disparity of the increase in BMD are the object of further research in different ethnic groups.

Two important facets of reducing fragility fractures are one to prevent falls and secondly making patients compliant with the drugs. If the drugs are not taken or improperly taken, then the purpose of treatment fails. It has long been known that compliance of osteoporosis medications is low [12–14]. It was anticipated that daily injections for 24 months will be difficult for patients, but Adachi et al. [15] reported the acceptance and compliance of teriparatide in the range of 82%–89%. Later, reports from different parts of the world dropped the initial enthusiasm. Tanaka et al. [16] reported that in their patients, compliance was lower to 61.0% at the end of the 1^st^ year. Hazel-Fernandez et al. [17] found that adherence was below par, and this was due to high costs of therapy. In our study, we found that adherence was 94.5%, which is the highest reported to date. There are a couple of reasons for this; since the medical care in Saudi Arabia is universal, the government pays for all care. Second, the department was dedicated to dealing with osteoporosis patients in a special way of checking them during treatment, and third, the Eli Lilly support group had provided a trained pharmacist to monitor and check on patient compliance.

Siris et al. [18] reported that the incidence of hip fractures in osteoporotic women was 26% and 18% in nonvertebral fractures, while Lou and his associates (2009) [19] found that in their patients aged >50 years, 23.3% fractures occurred in the spine and 21.6% in the femoral neck. Patients with osteoporosis have treatment options of taking antiresorptives or anabolics, and the goal remains to prevent or slow down bone loss and ultimately reduce the risk of fractures. In a recent trial in elderly patients, zoledronic acid, a strong intravenous antiresorptive agent, showed an increase in BMD in the spine and proximal femur, but the incidence of fractures did not positively change [20, 21]. In this study, 17 (4.09%) had fractures during the course of the treatment. This is lower than that reported in the Western literature. We believe this could be due to a couple of reasons. First, adherence was much higher as the treatment was provided free of charge by the government, monitoring was exceptional, and patients were seen every two months, and lastly, vitamin D and calcium levels were regularly checked, and a higher dosage of vitamin D of 4000 IU was given daily to all patients.

Our study has some limitations as there was no placebo or other drugs to compare with but the strength being a good number of patients in the study group and from a single center and meticulous follow up. In conclusion, the efficacy of teriparatide to increase the BMD in the range of 20% was much greater in the ME population when compared to the western reports, and adherence was higher than reported elsewhere. There was a robust decrease in the incidence of fresh fractures. We believe that higher compliance was important in achieving the results in this study.

In conclusion, this study shows that 94.5% patients completed the treatment duration. There was an increase in the BMD and a decrease in the T-scores of the lumbar spine and hip was significant so was the reduction in the number of fractures.

## Figures and Tables

**Table 1:** Frequency for prior treatment (*n* = 415).

Prior treatment	Frequency (n)	(%)
Yes	167	40.2
No	248	59.8
Total	**415**	**100.0**

Table 1 shows the presence of prior treatment in patients. The majority of patients (59.8%) have not undergone any prior treatment, while 40.2 percent of patients have undergone prior treatment.

**Table 2:** Difference in mean BMD g/cm^2^ and *T-*score in the hip and lumbar spine (L1-4) between pretest and post-test using the paired sample *t*-test (*n* = 415).

	Pre	Post	Mean difference (MD)	*P* value
	Mean ± SD			
Hip	−3.1 ± 0.79	−1.5 ± 0.62	−0.5464	0.001 ^*∗*^ ^*∗*^
Spine (L1-4)	−4.1 ± 0.86	−3.2 ± 0.87	−0.8612	0.001 ^*∗*^ ^*∗*^
Hip (BMDg/cm^2^)	0.781 ± 0.09	0.876 ± 0.07	0.95	0.001 ^*∗*^ ^*∗*^
Spine (BMDg/cm^2^)	0.505 ± 0.06	0.614 ± 0.11	0.109	0.001 ^*∗*^ ^*∗*^

^*∗*^ ^*∗*^*p* < 0.01.

## Data Availability

The data are available at the data repository: https://doi.org/10.6084/m9.figshare.21371781. The data are available under the terms of the Creative Commons Zero “No rights reserved” data waiver (CC0 1.0 Public domain dedication).
